# The Need for Improved Detection of Urinary Tract Infections in Young Children

**DOI:** 10.3389/fped.2017.00024

**Published:** 2017-02-21

**Authors:** Tracy E. Bunting-Early, Nader Shaikh, Lynn Woo, Christopher S. Cooper, T. Ernesto Figueroa

**Affiliations:** ^1^Outcomes Positive, Inc., Newark, DE, USA; ^2^Children’s Hospital of Pittsburgh, Pittsburgh, PA, USA; ^3^Rainbow Babies and Children’s Hospital, Cleveland, OH, USA; ^4^University of Iowa Children’s Hospital, Iowa City, IA, USA; ^5^Nemours/Alfred I. duPont Hospital for Children, Wilmington, DE, USA

**Keywords:** urinary tract infections, young children, detection, diagnosis, survey research, pediatricians, fever, prevalence

## Abstract

**Background and objectives:**

An estimated 400,000 urinary tract infections (UTIs) are diagnosed annually in children aged <3 years in the United States; yet >50% of febrile UTIs may be missed in this population. Here, we explored possible barriers to diagnosing febrile UTIs in very young children through social research of community pediatricians.

**Methods:**

Following qualitative interviews, a quantitative survey was developed that included a high-risk case for febrile UTI, presented before prompting for the topic of the survey, to gauge practice of delayed testing. Factors associated with delay were explored using univariate logistic regression. The final survey link was sent to three populations *via* email, with the largest response from a survey sent to pediatricians in Pennsylvania, which formed the basis of our primary results.

**Results:**

Of the 218 evaluable responses, 59.6% of physicians would initially test urine in the high-risk case patient, while 21.6% would choose to continue fever reducer and follow-up in 2 days. In the knowledge-based questions, 67.5, 34.0, and 35.6% of respondents identified the correct prevalences in total population, Caucasian girls, and uncircumcised boys, respectively. Many pediatricians (59.5%) believed that delays in detection are common in clinical practice. Physicians who chose to delay testing were more likely to be female, in practice for <25 years, to underestimate prevalence of febrile UTIs and have greater number of children seen per week (all *P* ≤ 0.02).

**Conclusion:**

Our findings support the need for improved communication and education about prevalence in higher risk populations, outcomes associated with delayed diagnosis, and optimal skills for collection of urine in young patients.

## Introduction

Urinary tract infections (UTIs) in very young children are relatively common and frequently present with non-specific symptoms, making timely diagnosis challenging and emphasizing the importance of health-care providers maintaining a high index of suspicion for UTI in this population (1–3). Overall prevalence of underlying UTIs in young children with fever have been shown to be 5–6%, with higher rates in Caucasian girls (16–17%) and uncircumcised boys (8%) (4, 5). The number of UTIs diagnosed annually in children under 3 years of age in the United States is estimated at 400,000 (6). Prospective studies have shown that diagnosis of up to 50–70% of febrile UTI cases may be missed, even in settings with high alert for UTIs or ample resources for testing (4, 7, 8).

Urinary tract infections in young children, particularly febrile UTIs that are significantly more likely to involve the kidney, may cause acute discomfort and permanent kidney scarring (9, 10). Delays in treatment may put young children at a greater risk of permanent kidney scarring (11), making the potential for missed infections in this population a significant health care concern worthy of further study.

Beyond the unavoidable limitations to diagnose all UTIs in this population, there may be barriers to detection that can be improved, such as increased awareness for which patients are at greater risk or improved access or techniques for obtaining urine samples. The American Academy of Pediatrics (AAP) Subcommittee on UTI requires urine culture for diagnosis of UTIs in this age group, with sample collected *via* urethral catheterization or suprapubic aspiration recommended (3). Clinical staff who are well trained and comfortable in performing these techniques may not be routinely available, particularly in smaller or community-based pediatric primary care settings.

No published studies have focused on the reasons for missed and/or delayed UTI diagnosis in young children. To evaluate the knowledge, attitudes, and practices of pediatricians, the present study explores the problem of missed/delayed diagnosis of febrile UTIs in very young children through qualitative research followed by quantitative surveys.

## Materials and Methods

### Study Plan

**Figure 1:**
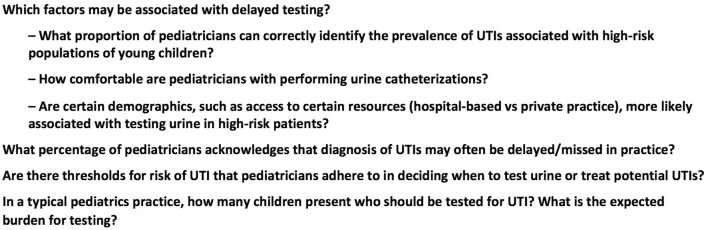
**Questions addressed in quantitative research**.

A survey development period of substantial qualitative research to explore potential barriers to diagnosis was followed by quantitative research performed *via* an online survey of pediatricians. Qualitative research consisted of in-person interviews with community-based primary care and specialist pediatricians, nurses, family practitioners, and parents/caregivers. Additionally, an online survey of parents/caregivers was conducted that informed on areas of concern from the patient perspective (12). Several potential barriers or gaps were identified that formed the basis of our quantitative survey (Figure 1).

A pilot survey was conducted *via* email to the Delaware Chapter of the AAP (*N* = 285) on December 4, 2014, ensured that the final study survey was optimized to facilitate responses, while remaining highly informative.

### Final Survey

**Figure 2:**
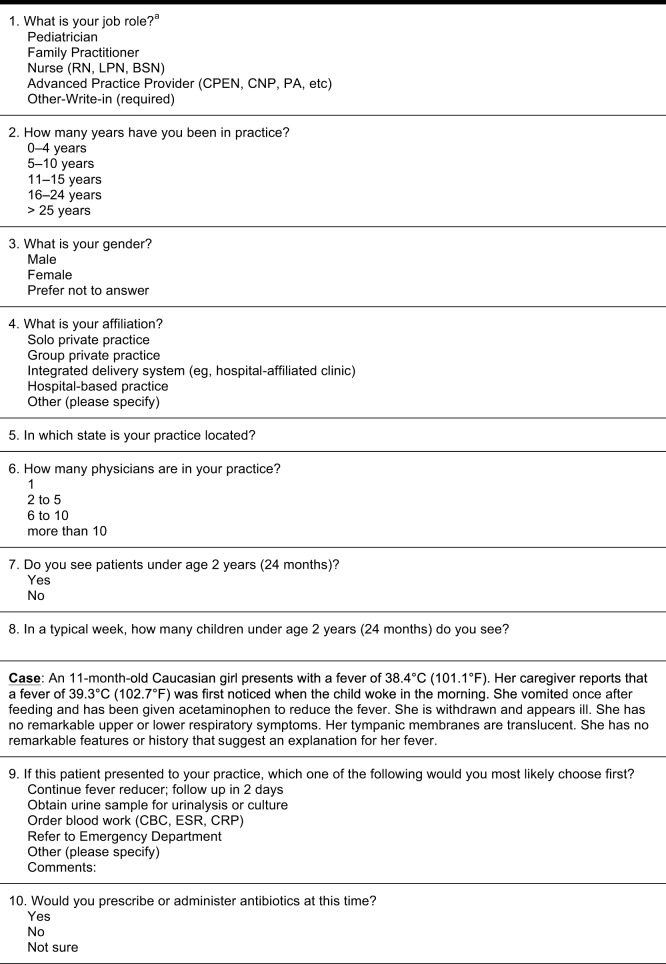

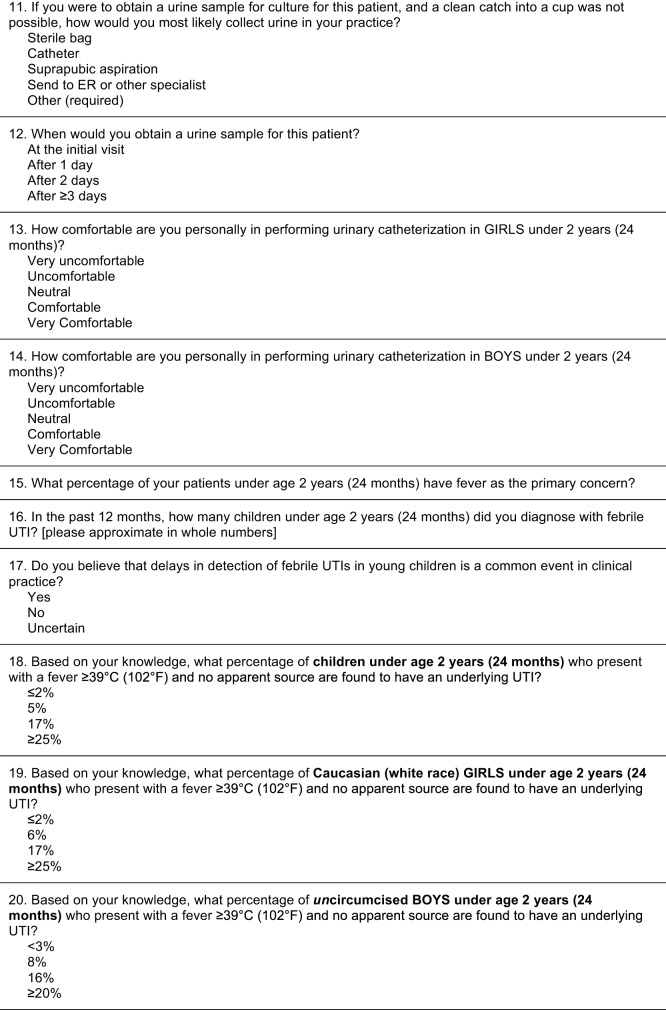

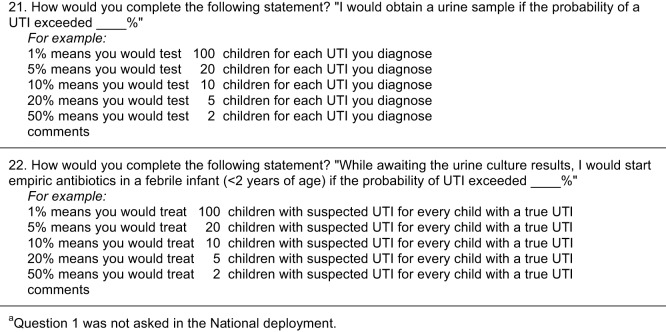
**Final quantitative survey**.

The final survey (21 items; Figure 2) was deployed to three distinct pediatrician samples. The Pennsylvania Survey was deployed to the email list of the Pennsylvania Chapter of the AAP and forms the basis for our primary study results presented here. Two previous deployments of the final survey, the National Survey [sent *via* email to a national sampling from the American Medical Association (AMA) contact list] and the Rainbow Babies Survey (sent *via* email to subscribers to the Rainbow Babies and Children’s Hospital in Cleveland, OH newsletter) did not yield robust sample numbers but are included to demonstrate how overall findings from the Pennsylvania survey extrapolate to other samples of physicians.

The Pennsylvania Survey was deployed (individual email containing a link) on October 5, 2015 (*N* = 2,183), with a follow-up reminder on October 9, 2015 (*N* = 2,172). The National Survey was sent in a single deployment (individual email containing a link) on June 20, 2015, to 5,111 pediatricians randomly pulled from the AMA national database. The Rainbow Babies Survey was deployed on August 18, 2015, as a link within the regularly scheduled “Rainbow Clinical Update” email newsletter (estimated 400–500 physician and non-physician recipients).

### Survey Execution

The pilot survey and final survey were hosted on the web-based software services provided by http://surveymonkey.com (SurveyMonkey Inc., Palo Alto, CA, USA) and http://surveygizmo.com (Widgix Software, LLC, Boulder, CO, USA), respectively. Completion of all survey questions was required, such that if a respondent did not want to answer a question, their response would terminate with their last completed question. Response choices were randomized, unless another logical, unbiased order was appropriate. This research was exempt from Institutional Review Board review because no identifiable information about the participants was recorded.

### Analysis of Survey Results

The case question of the survey provided an unprompted simulation of a real-life case scenario that was designed to quantify the practice of delayed testing of an ill, febrile child with a higher than average likelihood of UTI. The remaining questions were designed to correlate with this finding or add perspective to the knowledge, attitude, and practice in pediatric care, regarding the diagnosis of UTIs in very young children. For continuous variables, mean, median, minimum, and maximum are provided, and for categorical variables, numbers and percentages are provided. Demographic factors were assessed for association with choosing to delay urine testing using univariate logistic regression, with 0.05 as the level of significance.

Respondents who did not care for children under 2 years of age were excluded. Responses were excluded if the entry terminated before answering the case question or if it appeared to be a partial response followed by a more complete, duplicative response (same Internet Protocol address, very similar/identical answers, ≤1 h of each other). The remaining complete and partial responses were considered evaluable. Responses to individual questions were excluded when the answers were nonsensical (percentages with values over 100 or responses that included “?”). When responses were open ended and ranges were provided, an average was taken, e.g., “8–12” was resolved to “10.” Affiliations that were entered by respondents as “other” were reclassified based on likely setting, whereas resident, hospitalist, hospital plus outpatient, or university were classified as hospital based in our analysis. For calculating the average time to take the survey, only evaluated, full responses with completion times with duration <1 h were included.

## Results

### Survey Responses

The Pennsylvania Survey had 251 responses as a result of the initial (*n* = 154) and reminder (*n* = 97) emails, yielding an overall response rate of 11.5% (10.0% for evaluable responses). In total, there were 193 full evaluable responses, 25 partial evaluable responses, and 33 disqualified responses. Of those disqualified, 27 did not reach the case question, 6 were likely duplicates, and 3 did not see children under 2 years of age.

Respondents were pediatricians, including some residents (*n* ≥ 21) and subspecialists (*n* ≥ 10). Most were female (68.3%). The largest proportion had up to 4 years in practice (32.1%; 70/218; Table 1). The next largest proportion had over 25 years (26.6%; 58/218) in practice. Nearly half (47.7%) belonged to large practices (≥10 physicians). Of the 32.1% (70/218) who were office based, most (65/70) were in group private practice. All respondents were located within Pennsylvania except seven (3.2%), which were located in California (*n* = 2), Delaware (*n* = 1), Maryland (*n* = 1), New Jersey (*n* = 2), and “not in United States” (*n* = 1). The median time to complete the survey was 4.97 min. Characteristics of the other surveys, in comparison, are provided in Table 2.

**Table 1:** **Pennsylvania survey demographics**.

Physician demographics, *n* (%)	Total (*N* = 218)
Sex^a^	
Female	149 (68.7)
Male	68 (31.3)
Years in practice	
0–4	70 (32.1)
5–10	31 (14.2)
11–15	20 (9.2)
16–24	39 (17.9)
≥25	58 (26.6)
**Practice demographics**	
No. of physicians in practice, *n* (%)	
1	8 (3.7)
2–5	65 (22.5)
6–10	41 (18.8)
>10	104 (47.7)
Affiliation, *n* (%)	
Solo and group private practice^b^	70 (32.1)
Integrated delivery system	49 (22.5)
Hospital based	92 (42.2)
Other	7 (3.2)
No. of children <2 years seen each week^c^	
Mean	24.9
Median	20
[Min, max]	[1, 100]
Percent of children <2 years with fever as primary concern^d^	
Mean	30.9
Median	20.0
[Min, max]	[0, 90]
No. of girls <2 years diagnosed with febrile UTI^e^	
Mean	6.3
Median	4.0
[Min, max]	[0, 50]
No. of boys <2 years diagnosed with febrile UTI^f^	
Mean	1.6
Median	1
[Min, max]	[0, 60]

*UTI, urinary tract infection*.

*^a^One respondent preferred not to answer this question*.

*^b^Five respondents were solo private practice*.

*^c^217 respondents*.

*^d^204 respondents*.

*^e^207 respondents*.

*^f^202 respondents*.

**Table 2:** **Characteristics of survey deployments**.

Survey	Evaluable responses, *N*	Response rate (%)	Population description
Pennsylvania	218	11.5	AAP members of Pennsylvania chapter, most were female (68.3%), had 0–4 years (32.1%) or ≥25 years (26.6%) in practice, with the largest affiliation (42.2%) being hospital based

National	43	0.84	AMA registry of pediatricians representing 19 states, most (74.4%) were in private practice (group *n* = 24; solo *n* = 8), male (53.5%), and had been practicing ≥16 years (74.4%; 16–24: *n* = 12; ≥25: *n* = 20)

Rainbow Babies	30	~6.9	Hospital newsletter community, included family practitioners (*n* = 4) and nurses (*n* = 3), about half were in private practice (group: *n* = 12; solo: *n* = 2; integrated delivery system: *n* = 6; hospital based: *n* = 9). Most were female (73.3%) and had been in practice ≥16 years (56.7%; 16–24: *n* = 9; ≥ 25: *n* = 8)

*AAP, American Academy of Pediatrics; AMA, American Medical Association*.

### Pennsylvania Survey Case Question

Responses to the initial case question, general knowledge, and attitudes are provided in Table 3. Over half (59.6%) of physicians would initially test urine in the case patient, while 21.6% would choose to continue fever reducer and follow-up in 2 days. The remaining respondents would order blood work or refer to the emergency department or other (*n* = 16). Of those who chose “other,” their responses included testing urine (7/16), assessing other symptoms (6/16; neurological, ability to keep fluids down in office challenge, and immunization status), admit (1/16), and more detailed response related to following up later (2/16). Of the 47 respondents who chose to delay, 43 answered the later question asking when they would obtain a urine sample; 97.7% (42/43) also chose to delay ≥2 days, demonstrating good internal alignment with responses.

**Table 3:** **Pennsylvania survey responses**.

	Total, *n* (%)
Case: An 11-month-old Caucasian girl presents with a fever of 38.4°C (101.1°F). Her caregiver reports that a fever of 39.3°C (102.7°F) was first noticed when the child woke in the morning. She vomited once after feeding and has been given acetaminophen to reduce the fever. She is withdrawn and appears ill. She has no remarkable upper or lower respiratory symptoms. Her tympanic membranes are translucent. She has no remarkable features or history that suggests an explanation for her fever	
Question: If this patient presented to your practice, which one of the following would you most likely choose first? (*n* = 218)	
Continue fever reducer; follow-up in 2 days	47 (21.6)
Obtain urine sample for urinalysis or culture	130 (59.6)
Order blood work (CBC, ESR, CRP)	9 (4.1)
Refer to emergency department	16 (7.3)
Other (please specify)	16 (7.3)
Question: Would you prescribe or administer antibiotics at this time? (*n* = 218)	
Yes	7 (3.2)
No	173 (79.4)
Not sure	40 (18.3)
Question: If you were to obtain a urine sample for culture for this patient, and a clean catch into a cup was not possible, how would you most likely collect urine in your practice? (*n* = 207)	
Sterile bag	33 (15.9)
Catheter	167 (80.7)
Suprapubic aspiration	0
Send to ER or other specialist	6 (2.9)
Other (required)	1 (0.5)
Question: When would you obtain a urine sample for this patient? (*n* = 207)	
At the initial visit	144 (69.6)
After 1 day	6 (2.9)
After 2 days	30 (14.5)
After ≥3 days	27 (13.0)
**General knowledge^a^ (*n* = 194)**	
Probability of UTI in children <2 years of age with fever and no source	
≤2%	23 (11.9)
6%	108 (67.5)
17%	48 (24.7)
≥25%	15 (7.7)
Probability of UTI in Caucasian girls <2 years of age with fever and no source	
≤2%	24 (12.4)
6%	64 (33.0)
17%	66 (34.0)
≥25%	40 (20.6)
Probability of UTI in uncircumcised boys <2 years of age with fever and no source	
≤2%	86 (44.3)
8%	69 (35.6)
16%	23 (11.9)
≥20%	16 (8.3)
Attitudes	
Believe that delay in detection of febrile UTIs a common event in clinical practice (*n* = 194)	
Yes	116 (59.8)
No	37 (19.1)
Uncertain	41 (21.1)
Comfortable performing catheterization in girls (*n* = 207)	
Very uncomfortable	23 (11.1)
Uncomfortable	36 (17.4)
Neutral	39 (18.8)
Comfortable	57 (27.5)
Very comfortable	52 (25.1)
Comfortable performing catheterization in boys (*n* = 207)	
Very uncomfortable	22 (10.6)
Uncomfortable	32 (15.5)
Neutral	41 (19.8)
Comfortable	61 (29.5)
Very comfortable	51 (24.6)
Threshold required for testing, *n* = 193	
Mean	10.3%
Median	5%
[Min, max]	[0, 55]
Threshold required for treatment, *n* = 183	
Mean	25.8%
Median	20%
[Min, max]	[0, 80]

*^a^Overall prevalence of underlying UTIs in young children with fever are generally accepted to be 5–6%, with higher rates in Caucasian girls (16–17%) and uncircumcised boys (8%) (4, 5)*.

### Knowledge, Attitudes, and Practices

In the knowledge-based questions, 67.5, 34.0, and 35.6% of respondents identified the correct prevalences in total population, Caucasian girls, and uncircumcised boys, respectively. More than half of pediatricians (59.5%) believed that delays in detection of febrile UTIs are common in clinical practice. Pediatricians vary in their comfort with performing catheterizations, with about a quarter being “very comfortable.” When asked what threshold of probability would be needed for the physician to test urine for UTI, the median response was 5%. The median threshold associated with decision to treat was 20%. Several respondents elaborated that they would base their treatment threshold on urinalysis results.

### Factors Associated with Choosing to Delay Urine Testing

**Figure 3:**
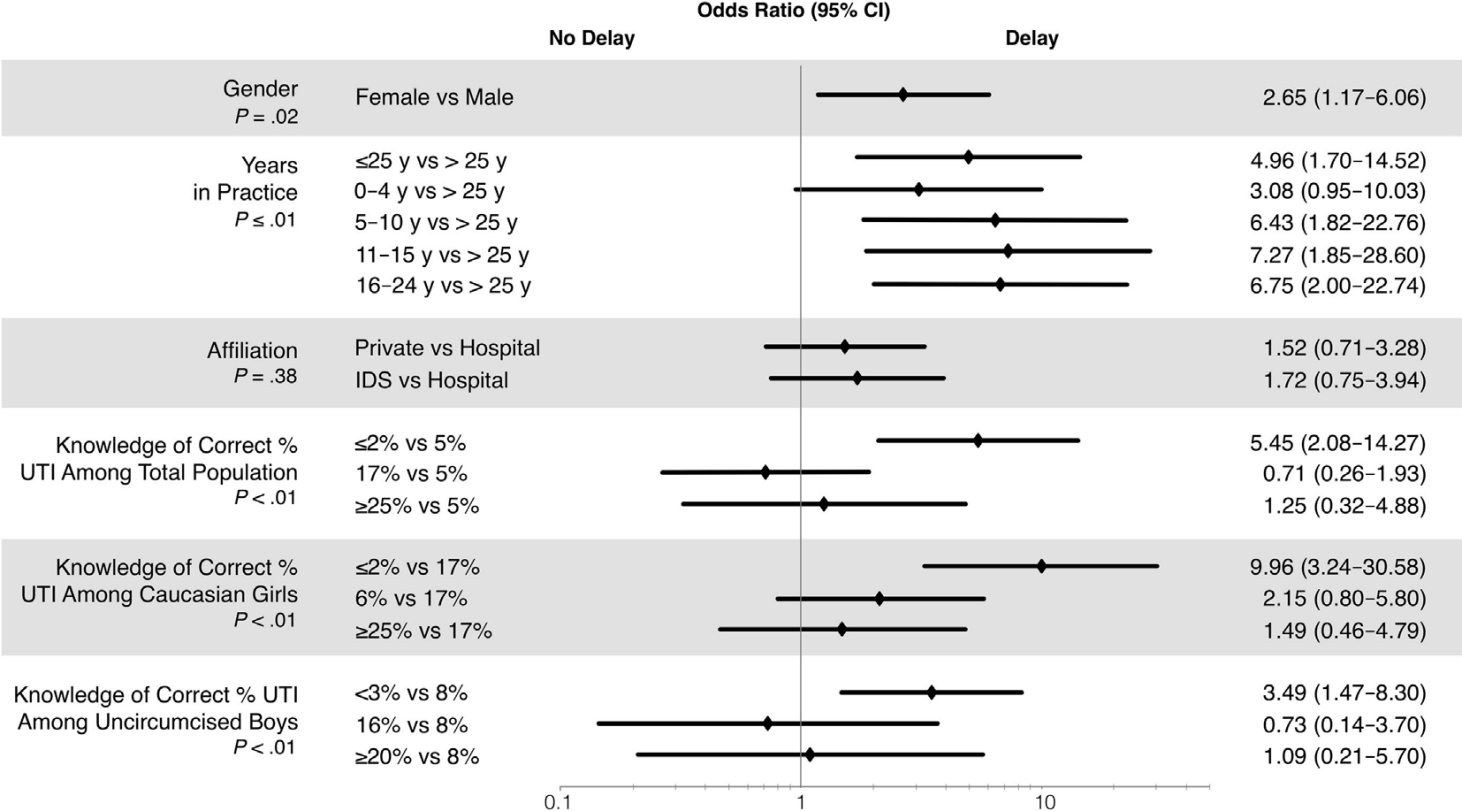
**Odds ratios correlation with the choice to delay urine testing in the case question**. IDS, integrated delivery system; UTI, urinary tract infection.

Several factors were significantly associated with choice to delay urine testing in the case question (Figure 3). Physicians who chose to delay testing were more likely to be female (*P* = 0.02), in practice for >4 years and <25 years (*P* = 0.01), and to underestimate prevalence of febrile UTIs (*P* < 0.01). Greater number of children seen per week increased the odds for choosing to delay (*P* = 0.01; OR 1.02, confidence limits 1.004–1.038), with the odds of choosing to delay increasing by 2% for each child seen per week. Hospital-based physicians chose to delay less often than those in private practice or integrated delivery systems, but the effect was not statistically significant (*P* = 0.38). None of the other factors tested were statistically significant.

### Generalizability of Pennsylvania Survey

**Figure 4:**
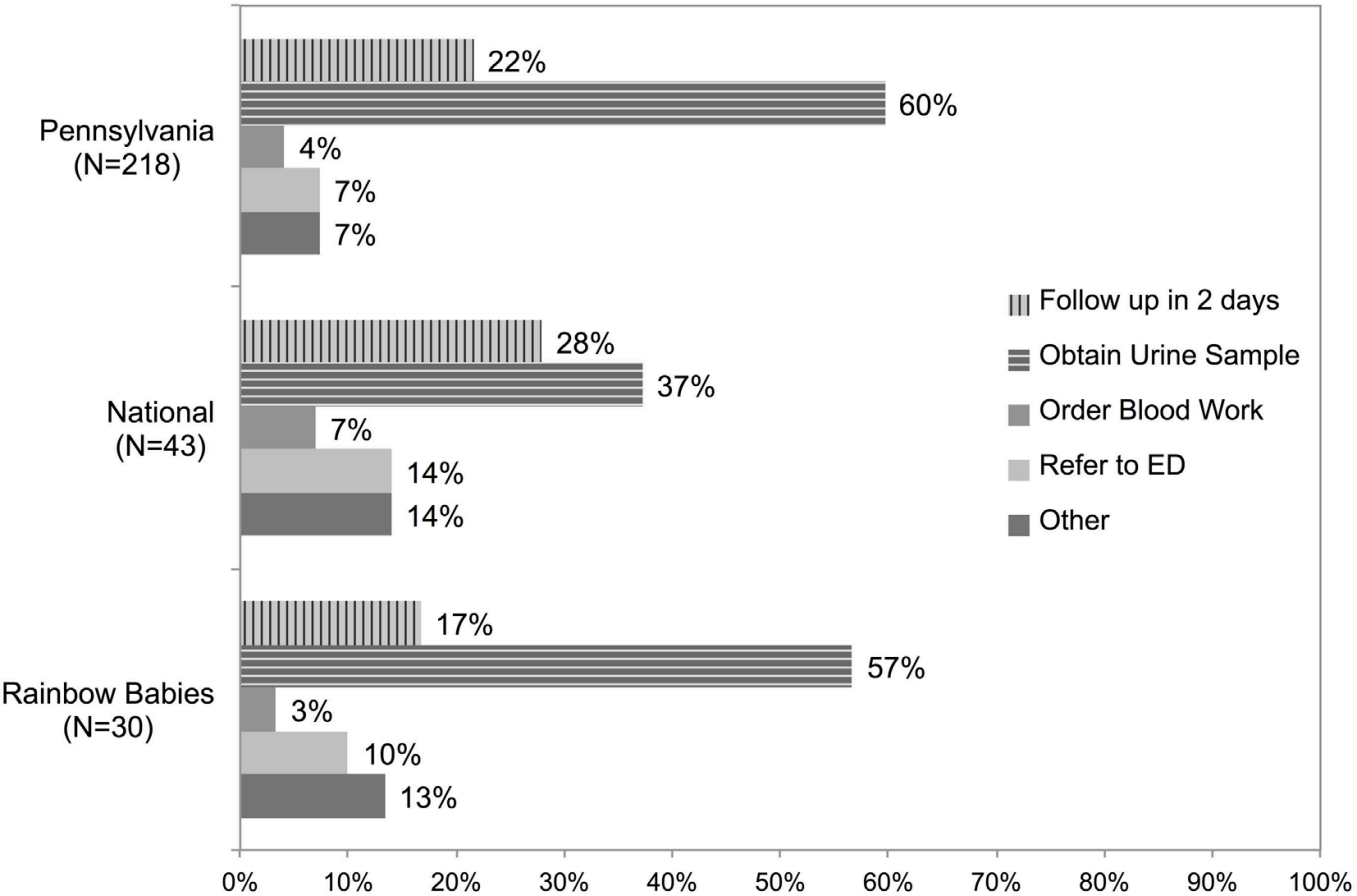
**Comparison of response to the first case question among deployments to Pennsylvania Chapter of the AAP, national sample of AMA pediatricians, and Rainbow Babies & Children’s Hospital newsletter**.

In comparison to the 22% of physicians in the Pennsylvania Survey who chose to delay, 17 and 28% chose to delay in the Rainbow Babies and National Surveys, respectively (Figure 4). The proportion of respondents who would test urine was similar in the Rainbow Babies and Pennsylvania Surveys (57% vs 60%), but much lower in the National Survey (37%). The proportions of physicians in the National Survey selecting the correct prevalences in the general knowledge questions were 39.0% (16/41), 31.7% (13/41), and 43.9% (18/41) for total population, Caucasian girls, and uncircumcised boys, respectively.

## Discussion

These results support the need to improve detection and diagnosis of UTI in young children. Approximately one-quarter to one-third of pediatricians would delay testing urine when presented with a case that had a one in six chance of underlying UTI. Similarly, one-third of pediatricians under- or overestimated the prevalence of underlying UTIs among the overall population under 2 years of age. Approximately two-thirds did not identify the correct prevalences for children at higher risk (Caucasian girls: 16–17%; uncircumcised boys: ~8%) and this shortcoming correlated with the choice to delay diagnostic testing. Moreover, a majority of pediatricians agreed that delay in detection of UTIs is a common event in clinical practice, and only about one-half of pediatricians acknowledged being comfortable or very comfortable in performing catheterizations in young children.

These findings are similar to results seen in both the National and Rainbow Babies Surveys and are consistent with observations reported by parents in a previous online survey (12). In the latter survey, ~45% of caregivers reporting that, in hindsight, they suspected previous illnesses may have been a kidney infection or UTI. Over half of the caregivers responding reported experiencing delays in having their child’s UTIs or kidney infections diagnosed. Just over half of parents reported that not all nurses/doctors seemed comfortable performing catheterization. Given the consistent findings across several samples, the opportunity for improvement in diagnosis of febrile UTIs in young children is most likely applicable to pediatric practice in general.

Missed infections and delays in diagnosis have a high potential for negative patient outcomes related to acute infection (i.e., sepsis) and longer term sequelae (i.e., renal scarring and insufficiency/failure). Long-term effects of renal scarring due to UTIs in very young children are difficult to appraise. No prospective trials have evaluated the impact of renal scarring over the lifetime of an individual. Prospective trials conducted in Sweden and Finland have only recently reported patient outcomes at 37–45 years of follow-up (9, 13). Those have shown that hypertension and kidney failure continue to emerge in some patients with kidney scarring due to UTIs in childhood (9, 13, 14). Therefore, the effects of missed infections may not be evident until much later in life but could conceivably cost those patients several years of dialysis and poor quality of life (3–5, 15–18).

Because our study is unique from past published research, we are unable to describe our findings as either an improvement or a decline in pediatrician knowledge, attitudes, or practices. Considering that either very experienced or newly graduated physicians were less likely to delay testing in our case, we cannot easily attribute the results to any single influence, such as guideline publication release, recent exposure to formal training, or evolution of other practice or health-care delivery patterns.

Recent efforts to improve the diagnosis of otitis media and reduce unnecessary antibiotic use (19) may uncover more frequent delays in UTI diagnosis and treatment in young children. Many experts believe that UTIs may be serendipitously treated when misdiagnosed as otitis media (8, 20). If true, raising the stringency on antibiotic treatment of otitis media without acknowledging the increased need for improved diagnosis of UTIs may therefore have an adverse outcome.

Possible explanations for lower than expected estimations for the prevalence of UTIs among pediatricians may be explained by lack of familiarity with the published research but also the possibility that missed UTIs are diagnosed and treated later by other health-care professionals, such as after-hours visits to urgent care centers or emergency departments. Also, since more patients are seeking alternative care options at urgent care centers away from traditional established single physician–patient relationships, detection of acute febrile UTI or patterns of recurrent infections may go unnoticed, as well as limiting the opportunities to correct one’s own practice.

A considerable limitation of this study was the apparent suboptimal response rate compared to other published rates of surveys in pediatricians (20–31%) (21, 22). Research aimed at understanding surveys of physicians reported a 7% response rate among pediatricians (survey deployment followed by a single email follow-up reminder) (23). Given this, our response rate of 11% seems reasonable.

The sampling of pediatricians from different email source lists inevitably exposes research to bias, which is one advantage of having sent to multiple sources for comparison. The AMA list represents all physicians, from which, pediatricians were selected; whereas the AAP chapter lists contained only AAP chapter members, who likely were required to pay annual dues.

Despite the current trend toward reducing testing and treatment across the health-care landscape, this research highlights the need for increased testing, education, and awareness that still exists in caring for preverbal febrile children. Our findings support the need for improved communication and education about prevalence in higher risk populations, outcomes associated with delayed diagnosis, and optimal skills for collection of urine in young patients. It also underscores a desire and need for innovative screening methods and less invasive testing.

The considerable rate of missed UTIs should also be accounted for when designing studies that attempt to assess children at the first febrile UTI by acknowledging the very real possibility that previous infections may have gone unrecognized. The inability of young patients to assist in the diagnosis of UTIs and the potential for later-in-life outcomes resulting from early-life care decisions support an err-on-the-side-of-caution approach. Great opportunity exists to help support health-care providers improve the diagnosis of UTIs in very young children in daily practice.

## Author Contributions

Conceptualized and designed the study: TB-E and TF. Data collection and coordination: TF, LW, and TB-E. Analysis supervision: NS and TB-E. Drafted initial manuscript: TB-E. Reviewed and revised manuscript, approved final manuscript as submitted: NS, LW, CC, TF, and TB-E.

## Conflict of Interest Statement

The authors have no potential conflicts of interest to disclose. Note that Dr Bunting-Early is currently employed by AstraZeneca Pharmaceuticals; however, Outcomes Positive and AstraZeneca have no mutual interests. And there are no potential conflicts of interest to report.

## Abbreviations

AAP, American Academy of Pediatrics; AMA, American Medical Association; UTI, urinary tract infection.
